# Effects of a monophasic combined oral contraceptive containing nomegestrol acetate and 17β-oestradiol compared with one containing levonorgestrel and ethinylestradiol on haemostasis, lipids and carbohydrate metabolism

**DOI:** 10.3109/13625187.2011.604450

**Published:** 2011-11-08

**Authors:** Ulla M Ågren, Marjatta Anttilat, Kristiina Mäenpää-Liukko, Maija-Liisa Rantala, Hilkka Rautiainen, Werner F Sommer, Ellen Mommers

**Affiliations:** * FSHS Kuopio, Kuopio, Finland and MSD, Espoo, Finland; † FSHS Tampere, Tampere, Finland; ‡ Turun Gynekologikeskus Ky, Turku, Finland; § Lääkärikeskus Adenova, Espoo, Finland; # Lääkärikeskus Gyneko Oy Oulu, Finland; ^ MSD, Oss, The Netherlands

**Keywords:** Oral contraceptives, Nomegestrol acetate, Oestradiol, Haemostasis, Lipids, Carbohydrates

## Abstract

**Objectives:**

To compare the effects of a combined oral contraceptive (COC) containing nomegestrol acetate and 17β-oestradiol (NOMAC/E2) on haemostasis, lipids, carbohydrate metabolism, C-reactive protein (CRP) and sex hormone-binding globulin (SHBG) with those of a COC containing levonorgestrel and ethinylestradiol (LNG/EE).

**Methods:**

In a randomised, open-label study, 121 healthy women, 18-50 years of age, were randomly assigned to receive NOMAC/E2 (2.5 mg/1.5 mg) in a 24/4-day regimen (*n* = 60) or LNG/EE (150 μg/30 μg) in a 21/7-day regimen (*n* = 61) for six cycles. The primary outcome was the change from baseline to cycle 6 for all indices.

**Results:**

All parameters were similar at baseline between the two groups. Over six cycles, NOMAC/E2 had less effect on most haemostatic indices than LNG/EE. Lipids were essentially unchanged with NOMAC/E2, whereas with LNG/EE high-density lipoprotein cholesterol decreased and low-density lipoprotein cholesterol and triglycerides slightly increased. NOMAC/E2 induced negligible changes in glucose and insulin parameters, in contrast to LNG/EE. A much smaller increase in CRP was observed with NOMAC/E2 than with LNG/EE. NOMAC/E2 was associated with a greater increase in SHBG.

**Conclusions:**

The monophasic COC NOMAC/E2 had less influence on haemostasis, lipids and carbohydrate metabolism than the COC LNG/EE.

## INTRODUCTION

Since their introduction 50 years ago, combined oral contraceptives (COCs) have become the most frequently used method of reversible contraception worldwide^1^. Over the past few decades, many advances have been achieved in COCs, including a reduction in the dose of the oestrogenic component ethinylestradiol (EE) and the introduction of more selective progestogens. This has resulted in improved tolerability^2^ and a more favourable cardiovascular risk profile^3^-^4^. The use of oestrogens and progestogens that are identical to or show greater resemblance with endogenous hormones represents the most recent advancement in COC development.

While the EE content in COCs is correlated with increased venous thromboembolism (VTE) risk, the influence of the progestogen component on VTE risk is controversial^5^. Although it has been suggested that some newer progestogens are associated with greater VTE risk^6^,^7^, the methodologies used in these studies have come into question^8^,^9^. Another study looked at confounders (weight, body mass index, smoking status, age, etc.) and found that COCs containing levonorgestrel (LNG) or norgestimate had similar VTE risk profiles compared with COCs containing gestodene or desogestrel^10^. Results from a post-marketing surveillance study (142,475 woman-years) that monitored cardiovascular outcomes among COC users (including those containing drospirenone [DRSP] or LNG) in Europe suggested that VTE risk was similar, independent of progestogen type^11^. Prescriber bias may also contribute to higher VTE rates, especially in smokers who may be more likely to receive prescriptions for COCs that contain newer progestogens^5^.

For several decades, researchers have been trying to incorporate the natural hormone 17β-oestradiol (E2), which is identical to the endogenous oestrogen, into COCs. Although many early formulations that contained E2 were associated with poor cycle control, which limited E2's clinical usefulness, initiatives to replace synthetic oestrogen with physiological hormone continued. It is still hypothesised that the use of E2 in combination with a suitable progestogen could further improve tolerability and minimise the deleterious effects of EE on hepatic metabolism^12^.

Recently, a monophasic COC containing nomegestrol acetate (NOMAC) in combination with E2 has been developed. NOMAC is a progestogen structurally similar to progesterone; it is devoid of oestrogenic, androgenic, glucocorticoid, and mineralocorticoid activity but displays an antioestrogenic activity on the endometrium and a moderate antiandrogenic activity^13^. Due to the enhanced selectivity profile of NOMAC, this COC may provide acceptable cycle control with putative neutral effects on cardiovascular or metabolic risks^14^.

The COC containing NOMAC/E2 (2.5 mg/1.5 mg) administered in a 24/4 (active/placebo)-day regimen provides effective suppression of ovarian activity and acceptable cycle control in women of childbearing age^15^,^16^. NOMAC/E2 has also demonstrated suppressive effects on the ovaries, cervical mucus and endometrium that are at least as strong as those of a comparator COC containing DRSP and EE^15^.

The current study was performed to assess the effects of NOMAC/E2 on haemostasis, lipids, carbohydrate metabolism, C-reactive protein (CRP), and sex hormone-binding globulin (SHBG). The influence of NOMAC/E2 on surrogate markers of adrenal and thyroid function, androgens, and androgen precursors was also evaluated, but will be reported separately^17^. LNG/EE (150 μg/30 μg) was used as a comparator based on regulatory guidance for safety evaluation of new COCs^18^.

## METHODS

The study was a randomised, open-label, comparative, parallel-design study conducted in five study centres in Finland. Advertisements were used to recruit women from the regions surrounding the study centres (Kuopio, Tampere, Turku, Espoo, and Oulu). Throughout the trial, study volunteers were compensated for their time, travel costs, and inconvenience associated with study visits.

The study complied with the ethical principles of the Declaration of Helsinki and the International Conference on Harmonisation (ICH) guideline for Good Clinical Practice. An independent Ethics Committee reviewed and approved the study protocol (NCT00511355) before the start of the study. All participants in the study provided written informed consent.

### Subjects

Healthy, sexually active women aged 18-50 years with a body mass index between 17-29 kg/m^2^ were eligible if they had no contraindications for the use of contraceptive steroids and had not taken any other hormonal treatment (except contraceptives) during the two months preceding screening. Other exclusion criteria included an abnormal cervical smear; a clinically relevant abnormal laboratory finding; breastfeeding; and use of liver-enzyme-inducing drugs, investigational drugs or pharmacological agents affecting the haemostatic system (e.g., vitamin K, nonsteroidal antiinflammatory drugs, including aspirin).

### Study design

The study consisted of one pre-treatment cycle (cycle 0), six 28-day treatment cycles (cycles 1-6), and a post-treatment follow-up. At the screening visit, subjects were evaluated to determine their eligibility and collect general baseline data. They were instructed to discontinue the use of hormonal contraceptives, if applicable, and to use condoms whenever necessary. The pre-treatment cycle visit took place in the second half of the first spontaneous menstrual cycle after the screening visit. During the pre-treatment cycle visit, blood was taken in a fasted state during the morning for baseline assessment of metabolic indices and an oral glucose tolerance test (OGTT) was performed.

Eligible women were randomised in a 1:1 ratio to receive the investigational COC containing 2.5 mg NOMAC and 1.5 mg E2 *or* the comparator COC containing 150 μg LNG and 30 μg EE. Randomisation was performed using blocks with randomly permuted block sizes and an interactive voice response system. Women were stratified by age class (i.e., 18—35 years and 36-50 years). For six consecutive 28-day cycles, the women took one tablet of the study medication orally at approximately the same time each day from day 1 to day 28. The NOMAC/E2 regimen comprised 24 days of active hormone pills followed by four days of placebo pills; the LNG/EE regimen comprised 21 days of active hormone pills followed by seven days of placebo pills. Blood collection for metabolic assessments and an OGTT were repeated between day 15 and day 21 of treatment cycles 3 and 6 when participants returned for clinic visits in a fasted state. A final visit was scheduled between 8 and 14 days after the last tablet was taken in cycle 6 or after early discontinuation of treatment for the gathering of general follow-up data.

### Laboratory measurements

All laboratory examinations were performed by the Bio Analytical Research Corporation (Ghent, Belgium).

#### Haemostasis

##### Thrombin turnover /fibrinolysis indices

Enzyme-linked immunosorbent assays (ELISAs) (Enzygnost* F 1 + 2 [monoclonal] assay, Dade Behring Marburg GmbH, Marburg, Germany) were performed to quantify pro-thrombin fragment 1+2. D-dimer was measured by the immunoturbidimetric assay STA® Liatest® D-DI on an STA Analyser (Diagnostica Stago, Asieres sur Seine, France).

##### Anticoagulatory indices

A thrombin generation test using the fluorescence-based Thrombinoscope technique (Thrombinoscope BV, Maastricht, The Netherlands) was performed to calculate the endogenous thrombin potential (ETP)-based activated protein C (APC) sensitivity ratio. The activated partial thromboplastin time (aPTT)-based APC sensitivity ratio was determined using the Coatest® APC™ Resistance test (Chromogenix - Instrumentation Laboratory SpA, Milan, Italy) with the STA Compact® (Diagnostica Stago). The activity of antithrombin III (ATIII) was measured by means of the colorimetric STA STAchrom ATIII assay using bovine thrombin in an STA Compact (Diagnostica Stago) and protein C activity by means of the enzymatic chromogenic STAchrom Protein C assay on an STA Analyser (Diagnostica Stago). The immunoturbidimetric assay STA Liatest Free Protein S was used on an STA Analyser (Diagnostica Stago) to measure free protein S antigen. Total protein S antigen was detected using the ELISA Asserachrom® Total Protein S kit (Diagnostica Stago).

##### Procoagulatory indices

Factor II activity, factorVII coagulant activity (factor VIIc) and activated factor VII (factor Vila) were measured using the commercial clotting assays/reagents STA-Deficient II, STA-Deficient VII, STAclot Vila-RTF, STA-Neoplastme CI + and the automated analyser STA Compact (Diagnostica Stago). Factor VIII activity was determined by measuring factor dilution activity using the STA Analyser (Diagnostica Stago).

#### Lipids

Serum levels of total cholesterol and triglycerides were determined enzymatically using cholesterol oxidase-phenol/aminophenazone (CHOD-PAP) and glycerol-3-phosphate oxidase (GPO)-PAP methods, respectively, on a Modular P Analyser (Roche Diagnostics GmbH, Mannheim, Germany). High-density lipoprotein cholesterol (HDL-C) values were measured using modified enzymatic procedures on a Modular P system; low-density lipoprotein cholesterol (LDL-C) values were estimated by the Friedewald equation (i.e., total cholesterol minus HDL-C minus very low-density lipoprotein cholesterol estimated as triglyceride level divided by five)^19^. Lipoprotein (a) and apolipoprotein A1 and B levels were assessed by immunoturbidimetry on a Modular P system (Roche Diagnostics).

#### Carbohydrate metabolism indices

For the OGTT, glucose and insulin levels were measured before (t=0) and at 30, 60, 90, 120 and 180 minutes after drinking a glucose solution (75 g/100 ml). Serum glucose levels were measured according to the hexokinase ultraviolet method on a Modular P system; serum insulin levels were estimated by chemiluminescence (CLIA test) using the automated immunochemiluminescence analyser Immulite® (DPC Biermann, Bad Nauheim, Germany). Ion-exchange high-performance liquid chromatography was performed using the automated analyser HA-8160 (A. Menarini Diagnostics S.r.l., Firenze, Italy) for the determination of haemoglobin type A_1c_ (HbA_1c_).

#### CRP and SHBG

Serum levels of CRP were obtained with the immunochemiluminescence analyser Immulite® (DPC Biermann, Bad Nauheim, Germany). The electrochemiluminescence immunoassay ECLIA (Roche Diagnostics) was used to measure serum levels of SHBG.

### Statistical analysis

The trial was set up to confirm the expected differences in changes from baseline between the treatment groups in several metabolic indices, especially prothrombin fragment 1+2, D-dimer and ATIII. In previous trials (unpublished data), standardised effect sizes (i.e., differences between the treatment groups in changes from baseline divided by the common standard deviation) were found to be in the range of approximately 0.6—1.0, depending on the parameter and the variability range. Conservatively assuming a standardised effect size of approximately 0.6 as a difference worthwhile to detect, a sample size of 42 evaluable subjects per treatment group was needed using a two-sided statistical test with 80% power and a significance level of 5%. Compensating for up to 20% premature discontinuations from treatment and accounting for a potential power loss due to the non-parametric analysis, in total 60 subjects were to be randomised per treatment group.

Analyses of all metabolic indices were performed for all randomised subjects who took at least one dose of study medication. For all parameters, median values and interquartile ranges at baseline (defined as the last measurement before administration of the first study medication) and treatment cycles 3 and 6 are presented, as well as changes from baseline to cycle 6 (primary outcome). Interquartile ranges were preferred over standard deviations in order to limit the influence of outliers on the variability estimates. If a value less than the lower limit of quantification (LLOQ) was observed for a metabolic variable, a value of 0.5 X LLOQ for the respective laboratory test was imputed. If a value above the upper limit of quantification (ULOQ) was reported, a value equal to the ULOQ of the respective laboratory test was used.

Glucose and insulin responses during the OGTT were evaluated as the area under the curve over three hours (AUC_3_), which were calculated according to the trapezoidal rule. A correction factor was used if the last blood sample was not drawn at the exact time of 180 minutes after glucose administration. Based on the calculated AUC_3_, the incremental AUC_3_ was defined as the difference between the AUC_3_ and the concentration before OGTT (fasting concentration) multiplied by three hours.

All statistical analyses of metabolic parameters used two-sided tests performed at the 5% error level. Each parameter was analysed using a stratified Wilcoxon rank sum test (Cochran-Mantel-Haenszel test) adjusted for age class, applied on the changes from baseline to cycle 6. No correction for multiplicity was applied.

## RESULTS

**Figure 1:**
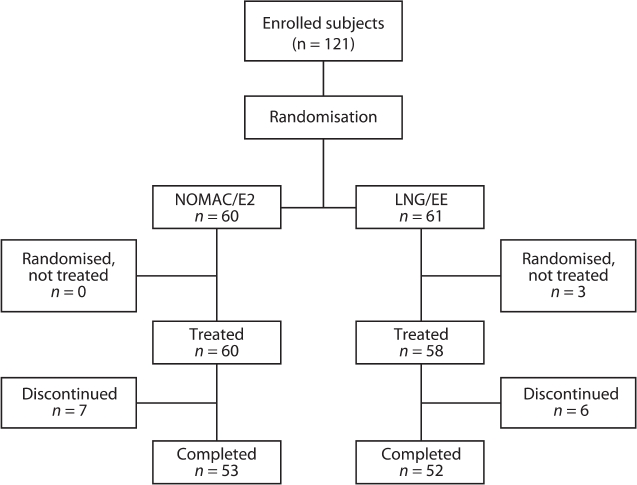
Disposition of subjects. NOMAC/E2, nomegestrol acetate/17β-oestradiol; LNG/EE, levonorgestrel/ethinylestradiol.

A total of 121 women were randomised to receive either NOMAC/E2 or LNG/EE (Figure 1). All 60 women in the NOMAC/E2 group received treatment; three of the 61 women in the LNG/EE group did not receive treatment because of a pre-treatment adverse event (AE) ('acne'), withdrawn consent or other personal reasons (found a new job). Seven women (11.7%) in the NOMAC/E2 group and six women (10.3%) in the LNG/EE group discontinued the treatment prematurely. Of these 13 discontinuers, eight women (four in each group) discontinued due to an AE, one due to pregnancy wish, one moved to another city, and three women were lost to follow-up. Overall tablet intake compliance was high in both treatment groups: 93.1% and 87.7% of women in the NOMAC/E2 and LNG/EE groups, respectively, took the daily tablet on at least 95% of treatment days.

The demographic and clinical characteristics were similar between the two treatment groups at baseline (Table 1), with the exception of smoking prevalence, which was higher among women in the NOMAC/E2 group. Baseline values for all metabolic indices in the two treatment groups were also similar (Tables 2-4).

**Table 1:** Subject characteristics at screening.

	*NOMAC/E2 (n=60)*	*LNG/EE (n=58)*	*Total (n = 118)*
Age (years), mean (SD)	28.2 (8.2)	29.1 (78)	28.7 (8.0)
Race, *n* (%)			
Black	1 (1.7)	0	1 (0.8)
White	59 (98.3)	58 (100.0)	117 (99.2)
Weight (kg), mean (SD)	62.8 (9.7)	61.7 (9.0)	62.3 (9.3)
BMI (kg/m^2^), mean (SD)	23.0 (2.9)	22.3 (2.5)	22.7 (2.7)
Nulligravida, *n* (%)	35 (58.3)	34 (58.6)	69 (58.5)
Parity*, *n* (%)			
NA (0 without pregnancy)	35	34	69
0 (with pregnancy)	4 (16.0)	3 (12.5)	7(14.3)
1	7 (28.0)	6 (25.0)	13 (26.5)
≥2	14 (56.0)	15 (62.5)	29 (59.2)
Last contraceptive method used within three months prior to screening**, *n* (%)			
None	1 (1.7)	4 (6.9)	5 (4.2)
Combined oral contraceptive	19 (31.7)	22 (37.9)	41 (34.7)
Progestogen-only-pill	3 (5.0)	1 (1.7)	4 (3.4)
ILJD (hormonal)	5 (8.3)	1 (1.7)	6(5.1)
ILJD (non-hormonal)	1 (1.7)	2 (3.4)	3 (2.5)
Vaginal ring or transdermal patch	5 (8.3)	7(12.1)	12 (10.2)
Foam, condom, suppositories, diaphragm	26 (43.3)	21 (36.2)	47 (39.8)
Smoking status			
Smokers, *n* (%)	14 (23.3)	7(12.1)	21 (178)
Non-smokers, *n* (%)	46 (76.7)	51 (879)	97 (82.2)

NOMAC/E2, nomegestrol acetate/17β-oestradiol; LNG/EE, levonorgestrel/ethinylestradiol; SD, standard deviation; BMI, body mass index; NA, not applicable; ILJD, intrauterine device.

* Gestational age ≥ 28 weeks;

** Single most important contraceptive method.

**Table 2:** Comparison of the effects of nomegestrol acetate/17β-oestradiol (N0MAC/E2) and levonorgestrel/ ethinylestradiol (LNG/EE) on thrombin turnover/fibrinolysis indices, anticoagulatory factors, and procoagulatory factors (medians [interquartile ranges]).

*Thrombin turnover/fibrinolysis indices*						

Treatment	*Baseline*	*Cycle 3*	*Cycle 6*	*Change from baseline to cycle 6*	*% change from baseline to cycle 6*	*p*-value*
Prothrombin fragments 1 + 2 (nmol/l)						
NOMAC/E2	0.15 (0.07)	0.15 (0.08)	0.14 (0.10)	−0.00 (0.06)	−1.7 (46.0)	0.085
LNG/EE	0.17 (0.10)	0.21 (0.12)	0.21 (0.12)	0.02 (0.10)	13.5 (59.6)	
D-dimer (mg/l FEU)						
NOMAC/E2	0.11 (0.19)	0.11 (0.16)	0.11 (0.12)	0.00 (0.00)	0.0 (0.0)	†
LNG/EE	0.11 (0.15)	0.22 (0.26)	0.11 (0.22)	0.00 (0.17)	0.0 (68.0)	
Anticoagulatory factors						
ETP-based APC sensitivity ratio						
NOMAC/E2	0.70 (0.40)	1.10 (0.60)	1.10 (0.40)	0.40 (0.60)	60.0 (80.0)	< 0.001
LNG/EE	0.70 (0.60)	2.20 (1.00)	1.90 (1.25)	1.20 (1.00)	146.4 (160.0)	
aPTT-based APC) sensitivity ratio						
NOMAC/E2	1.00 (0.20)	0.98 (0.17)	1.05 (0.16)	0.03 (0.17)	3.3 (16.4)	0.97
LNG/EE	0.99 (0.14)	0.99 (0.13)	1.02 (0.13)	0.02 (0.15)	2.0 (14.9)	
Antithrombin III (%)						
NOMAC/E2	99.5 (13.5)	100.0 (15.0)	103.0 (12.0)	4.0 (14.0)	3.9 (13.4)	0.004
LNG/EE	99.0 (15.0)	95.5 (10.0)	95.5 (16.5)	−3.5 (14.0)	−3.6 (14.1)	
Protein C (U/l)						
N0MAC/E2	105.5 (175)	101.0 (19.0)	104.0 (29.0)	−3.0 (19.0)	−3.1 (18.3)	0.002
LNG/EE	102.5 (25.0)	111.0 (24.0)	113.0 (25.5)	8.5 (20.0)	8.2 (20.2)	
Free protein S (%)						
N0MAC/E2	84.0 (20.5)	96.0 (22.0)	99.0 (33.0)	11.0 (18.0)	13.3 (21.0)	0.97
LNG/EE	85.0 (18.0)	93.5 (22.0)	102.0 (24.5)	11.5 (21.5)	11.9 (25.9)	
Total protein S (%)						
N0MAC/E2	79.0 (13.5)	85.0 (170)	83.0 (170)	4.0 (16.0)	4.7 (20.5)	< 0.001
LNG/EE	78.0 (14.0)	74.5 (12.0)	75.0 (11.5)	−3.0 (14.0)	−3.6 (175)	

***Procoagulatory factors***						

Factor II (%)						
N0MAC/E2	93.0 (15.0)	970 (14.0)	95.0 (19.0)	−1.0 (18.0)	−0.9 (18.5)	0.50
LNG/EE	94.5 (15.0)	101.5 (14.0)	95.0 (20.0)	3.0 (19.5)	3.0 (22.7)	
Factor VIla (U/l)						
N0MAC/E2	80.0 (41.5)	84.0 (44.0)	82.0 (470)	70 (46.9)	8.8 (63.3)	0.42
LNG/EE	71.0 (35.0)	91.5 (45.0)	86.0 (375)	9.0 (30.0)	14.4 (42.0)	
Factor Vile (%)						
N0MAC/E2	102.0 (30.5)	105.0 (36.0)	103.0 (28.0)	1.0 (33.0)	1.0 (33.3)	0.001
LNG/EE	101.0 (30.0)	98.5 (25.0)	94.5 (31.5)	−13.0 (275)	−12.7 (24.1)	
FactorVIII (%)						
N0MAC/E2	870 (45.0)	86.0 (45.0)	85.0 (45.0)	3.0 (470)	4.8 (51.8)	0.38
LNG/EE	88.5 (42.0)	870 (41.0)	95.5 (43.0)	6.0 (34.5)	6.8 (40.6)	

FEU, Fibrinogen equivalent units; ETP endogenous thrombin potential; aPTT activated partial thromboplastin time;

APC, activated protein C; Factor Vila, activated Factor VII; factor Vile, coagulated activated Factor VII.

* The Cochran-Mantel-Haenszel test adjusted for age class using standardised midranks, applied on the absolute changes from baseline to cycle 6. A *p*-value ≤ 0.05 indicates that the difference from baseline to cycle 6 (NOMAC/ E2 vs LNG/EE) was statistically significant.

† The *p*-value is not presented because > 50% of subjects had D-dimer values below the lower limit of quantification (LLOQ) of 0.22 mg/l FEU.

### Haemostasis

**Figure 2:**
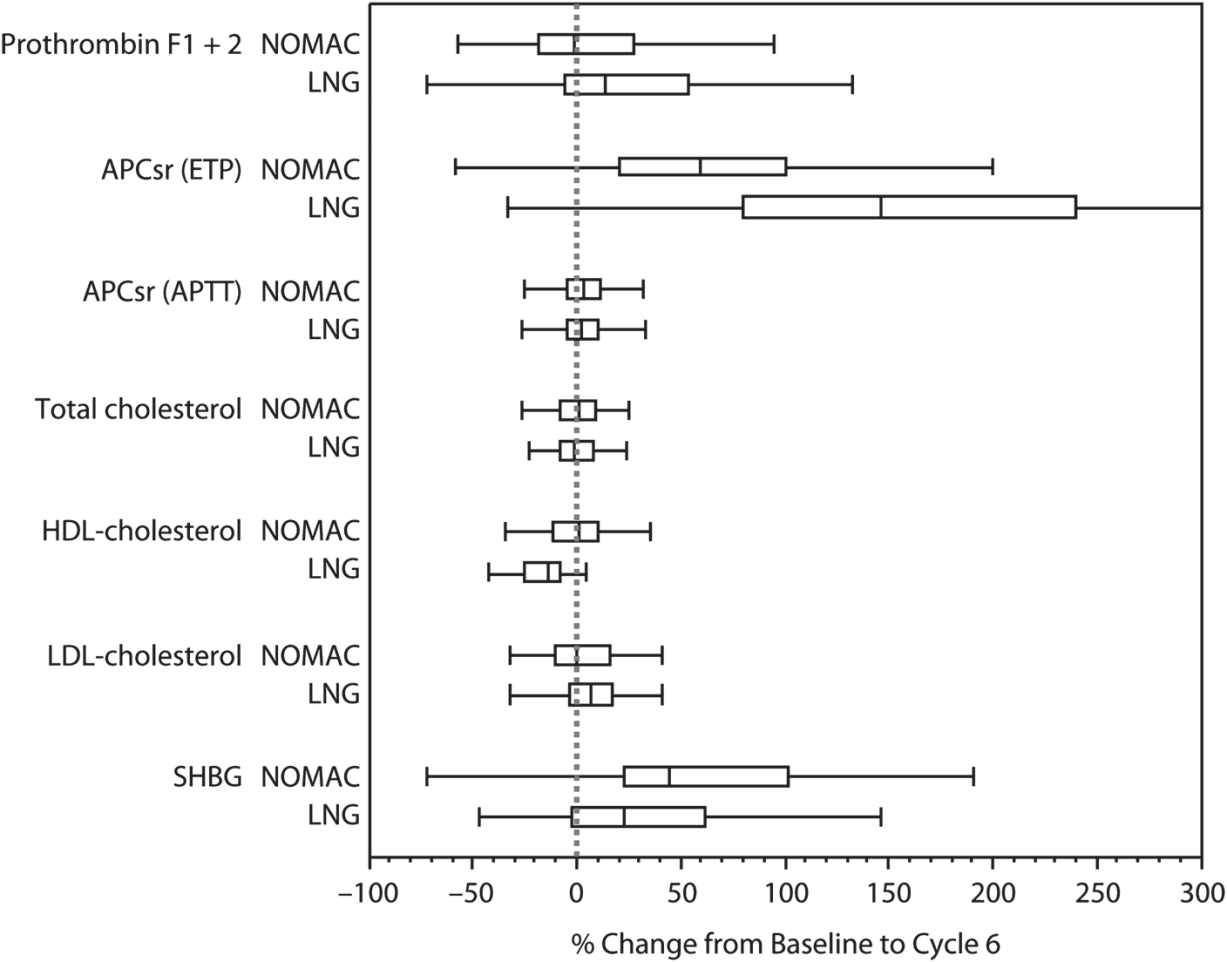
Box whisker plots for NOMAC/E2 and LNG/EE of relative changes from baseline to end of cycle 6 in selected haemostatic and lipid parameters and SHBG. The edges of the boxes present the 25th and 75th sample percentiles (quartiles). The distance between these sample percentiles is the interquartile range. The vertical line in the box shows the median and the whiskers are drawn up to the smallest and largest value within 1.5-times the interquartile range. NOMAC, nomegestrol acetate/17β-oestradiol; LNG, levonorgestrel/ethinylestradiol; APCsr, activated protein C sensitivity ratio; ETP, endogenous thrombin potential; aPTT, activated partial thromboplastin time; HDL-C, high-density lipoprotein cholesterol; LDLC, low-density lipoprotein cholesterol; SHBG, sex hormone-binding globulin.

Prothrombin fragments 1+2 essentially did not change in the NOMAC/E2 group, whereas a small increase in the LNG/EE group was observed (*p* = 0.085; Table 2; Figure 2). Although D-dimer was nearly unchanged in both groups, these results are inconclusive because more than 50% of the values were below the limit of detection.

The ETP-based APC sensitivity ratio increased by a statistically significant margin from baseline to cycle 6 in both groups; however, the change was much greater with LNG/EE than with NOMAC/E2 (*p*< 0.001; Table 2; Figure 2). The aPTT-based sensitivity ratio was nearly unchanged in both groups. Small changes from baseline were observed in the anticoagulatory factors ATIII, protein C, and free and total protein S in both treatment groups (Table 2), but the between-group differences for these parameters were found to be statistically significant (*p*< 0.01; Table 2) except for free protein S.

NOMAC/E2 and LNG/EE induced minimal changes from baseline in procoagulatory factors II, Vila and VIII (Table 2). For factor VIIc, a minimal change from baseline was observed in the NOMAC/ E2 group, whereas a decrease was observed in the LNG/EE group (*p* = 0.001; Table 2).

### Lipids

No clinically relevant changes were seen in total cholesterol, HDL-C, LDL-C, or total triglycerides among women receiving NOMAC/E2 during six treatment cycles (Table 3; Figure 2). LNG/EE treatment did not change total cholesterol either, but it decreased HDL-C, increased LDL-C, and increased total triglycerides. The decrease from baseline in HDL-C and the increase from baseline in LDL-C and total triglycerides observed in the LNG/EE group were statistically significantly different from changes observed in the NOMAC/E2 group (*p*< 0.05; Table 3). No changes in lipoprotein (a) were observed in the NOMAC/E2 group, and small changes were observed in the LNG/EE group, the difference being statistically significant (p < 0.001). Women in the NOMAC/E2 group had a significantly greater increase from baseline in apolipoprotein Al (*p* = 0.006) and a significantly smaller increase in apolipoprotein B (*p* < 0.001) than those in the LNG/EE group.

**Table 3:** Comparison of the effects of nomegestrol acetate/17β-oestradiol (N0MAC/E2) and levonorgestrel/ ethinylestradiol (LNG/EE) on lipid indices, CRR and SHBG (medians [interquartile ranges]).

*Lipid indices*						

*Treatment*	*Baseline*	*Cycle 3*	*Cycle 6*	*Change from baseline to cycle 6*	*% change from baseline to cycle 6*	p-value*
Total cholesterol (mmol/l)						
NOMAC/E2	4.33 (1.23)	4.22 (1.04)	4.35 (1.09)	0.08 (0.65)	1.3 (16.9)	0.69
LNG/EE	4.43 (1.01)	4.37 (0.91)	4.40 (1.14)	−0.03 (0.75)	−0.7 (15.8)	
HDL-C (mmol/l)						
NOMAC/E2	1.58 (0.41)	1.58 (0.44)	1.63 (0.44)	0.02 (0.32)	1.6 (21.0)	< 0.001
LNG/EE	1.66 (0.42)	1.47 (0.37)	1.35 (0.34)	−0.23 (0.32)	−13.1 (18.0)	
LDL-C (mmol/l)						
NOMAC/E2	2.28 (0.92)	2.07 (0.91)	2.15 (0.78)	−0.02 (0.48)	−0.5 (26.2)	0.046
LNG/EE	2.41 (0.81)	2.43 (0.98)	2.43 (1.09)	0.11 (0.52)	6.8 (20.1)	
Total triglycerides (mmol/l)						
NOMAC/E2	0.90 (0.32)	0.88 (0.47)	0.92 (0.41)	0.06 (0.29)	75 (34.8)	0.008
LNG/EE	0.75 (0.32)	0.93 (0.49)	0.99 (0.48)	0.15 (0.44)	170 (56.4)	
Lipoprotein (a) (g/l)						
NOMAC/E2	0.10 (0.15)	0.10 (0.16)	0.10 (0.19)	0.00 (0.03)	0.0 (20.3)	< 0.001
LNG/EE	0.11 (0.19)	0.10 (0.13)	0.10 (0.18)	−0.02 (0.05)	−14.0 (30.7)	
Apolipoprotein A1 (g/l)						
NOMAC/E2	1.59 (0.30)	1.66 (0.42)	1.73 (0.44)	0.20 (0.30)	12.1 (20.2)	0.006
LNG/EE	1.56 (0.32)	1.65 (0.35)	1.66 (0.30)	0.09 (0.26)	4.6 (175)	
Apolipoprotein B (g/l)						
NOMAC/E2	0.61 (0.21)	0.60 (0.24)	0.62 (0.26)	0.03 (0.12)	4.6 (20.0)	< 0.001
LNG/EE	0.63 (0.16)	0.77 (0.27)	0.79 (0.28)	0.16 (0.21)	28.0 (32.0)	
CRP (mg/l)						
NOMAC/E2	0.40 (0.60)	0.60 (1.40)	0.70 (1.00)	0.20 (0.80)	66.7 (200.0)	< 0.001
LNG/EE	0.40 (1.30)	1.75 (3.00)	1.70 (2.85)	0.95 (2.20)	258.3 (495.5)	
SHBG (nmol/l)						
NOMAC/E2	65.5 (33.6)	96.3 (55.5)	1079 (578)	33.8 (52.0)	44.1 (78.8)	0.019
LNG/EE	76.5 (40.6)	93.9 (476)	98.3 (30.8)	15.3 (42.6)	22.4 (64.1)	

HDL-C, high-density lipoprotein cholesterol; LDL-C, low-density lipoprotein cholesterol; CRR C-reactive protein; SHBG, sex hormone-binding globulin.

* The Cochran-Mantel-Haenszel test adjusted for age class using standardised midranks, applied on the absolute changes from baseline to cycle 6. A *p*-value ≤ 0.05 indicates that the difference from baseline to cycle 6 (NOMAC/ E2 vs LNG/EE) was statistically significant.

### Carbohydrate metabolism

Over six cycles of treatment, women receiving NOMAC/E2 had negligible changes from baseline in the AUC_3_ and incremental AUC_3_ for both glucose and insulin (Table 4). In the LNG/EE group, increases from baseline were observed for all four parameters. The differences between treatment groups were statistically significant for all of these indices (*p*≤ 0.002; Table 4). No changes in HbA_1c_ were observed in either group.

**Table 4:** Comparison of the effects of nomegestrol acetate/17β-oestradiol (N0MAC/E2) and levonorgestrel/ ethinylestradiol (LNG/EE) on carbohydrate indices (medians [interquartile ranges]).

*Carbohydrate indices*						

*Treatment*	*Baseline*	*Cycle 3*	*Cycle 6*	*Change from baseline to cycle 6*	*% change from baseline to cycle 6*	p-value*
AUC_3_ for glucose (hours X mmol/l)						
N0MAC/E2	15.73 (3.49)	15.95 (3.46)	15.31 (3.75)	0.79 (3.37)	6.1 (25.7)	0.002
LNG/EE	14.12 (3.26)	16.19 (4.58)	16.34 (4.71)	2.08 (3.16)	13.9 (24.5)	
Incremental AUC_3_ for glucose (hours X mmol/l)						
N0MAC/E2	1.15 (3.35)	1.27 (3.29)	1.25 (2.93)	0.61 (3.02)	25.4 (2076)	< 0.001
LNG/EE	0.64 (3.06)	2.95 (4.05)	2.87 (4.03)	2.07 (3.50)	56.5 (273.0)	
AUC_3_ for insulin (hours X mmol/l)						
N0MAC/E2	585.6 (304.0)	654.5 (488.3)	620.3 (3571)	−4.1 (240.6)	−0.9 (33.1)	< 0.001
LNG/EE	540.6 (218.7)	702.6 (375.0)	704.7 (356.0)	1779 (275.2)	26.4 (53.7)	
Incremental AUC_3_ for insulin (hours X mmol/l)						
N0MAC/E2	4476 (302.3)	496.1 (3873)	481.1 (270.5)	−29.5 (241.3)	−6.1 (571)	0.002
LNG/EE	432.1 (179.2)	592.8 (348.5)	588.3 (284.7)	1479 (299.8)	35.9 (69.5)	
HbA_1c_ (%)						
N0MAC/E2	5.35 (0.35)	5.40 (0.30)	5.30 (0.30)	0.00 (0.20)	0.0 (3.7)	0.37
LNG/EE	5.30 (0.20)	5.40 (0.20)	5.30 (0.30)	0.00 (0.20)	0.0 (3.7)	

AUC_3_, area under the curve over three hours; HbA_1c_, haemoglobin type A_1c_.

* The Cochran-Mantel-Haenszel test adjusted for age class using standardised midranks, applied on the absolute changes from baseline to cycle 6. A *p*-value ≤ 0.05 indicates that the difference from baseline to cycle 6 (NOMAC/ E2 vs LNG/EE) was statistically significant.

### CRP and SHBG

Although CRP concentrations increased after treatment in both groups, the increase from baseline was significantly smaller with NOMAC/E2 (+ 67%) than with LNG/EE (+ 258%) (*p* < 0.001; Table 3). Both COCs were associated with increases in SHBG concentrations, with a significantly greater increase in the NOMAC/E2 group (44%) compared with the LNG/ EE group (22%) (*p* = 0.019; Table 3; Figure 2).

### Contraceptive efficacy and tolerability

No pregnancies occurred during the trial in either treatment group. NOMAC/E2 was generally well tolerated, with a similar AE profile as LNG/EE. AEs reported with an incidence ≥ 5% (in any treatment group) in women who received either NOMAC/E2 or LNG/EE, respectively, were upper respiratory tract infection (6 and 5 subjects),headache (3 and 7 subjects), acne (2 and 4 subjects), influenza (1 and 4 subjects), metrorrhagia (3 and 1 subject), and vaginal candidiasis (3 and 1 subject). One serious adverse event (SAE), worsening of a congenital mitral valve leak, was reported in the NOMAC/E2 group. The subject was withdrawn from the study. No SAEs were reported in the LNG/EE group. Eight women (four women in each group) discontinued treatment during the study because of an AE. In the NOMAC/E2 group, reasons for discontinuation due to an AE included depression and nausea; one subject experienced a combination of tachycardia, pain in the calf, and weakness in the limb. In the LNG/EE group, AEs that led to discontinuation included decreased sexual desire, nausea, and headache.

## DISCUSSION

This randomised study compared the effects of NOMAC/E2 administered in a 24-day regimen and LNG/EE in a 21-day regimen on metabolic indices. After six cycles of treatment, NOMAC/E2 had minimal influence on parameters related to haemostasis, lipids, and carbohydrate metabolism. Overall, NOMAC/E2 had less effect on these indices than the comparator LNG/EE. Both NOMAC/E2 and LNG/ EE were associated with moderate increases in SHBG and increases in CRP. Changes in CRP were particularly marked with LNG/EE.

An estimated 100 million women worldwide use COCs^1^, which have demonstrated an excellent overall contraceptive efficacy and safety since their introduction in 1960. However, epidemiological evidence linking COC use with an increased risk of cardiovascular morbidity, particularly of VTE, remains a cause for concern. Several factors have been associated with VTE risk in women taking EE-containing COCs, including the EE dose^20^,^21^ and type of progestogen used^21^-^25^. Whether observed differences in VTE risk among COC formulations can be explained by confounding factors and biases in study design and methodology remains the subject of debate^5^. Reductions in the dose of EE (< 50 μg) have resulted in a decreased incidence of VTE^21^, ischaemic stroke^25^,^26^, and myocardial infarction^25^. Further reduction of the EE dose to 20 μg may further decrease the VTE incidence (suggested in a case-control study^21^), but the evidence for this is limited. Even very low doses of EE (i.e., 10 μg) have been shown to adversely affect haemostatic parameters^27^.

COC formulations containing oestrogen that are structurally similar or identical to endogenous E2 have been sought for several decades because they are expected to offer an improved safety profile. A number of clinical studies have demonstrated that E2 and its derivative oestradiol valerate have less effect on hepatic protein synthesis than EE and have more favourable lipid and haemostatic profiles^27^-^29^. This is confirmed by data from the present study, in that NOMAC/E2 had less influence on indices of haemostasis, lipid and carbohydrate metabolism than LNG/EE (150 μg/30 μg), as well as by the results of a previous study, which showed that NOMAC/E2 induced smaller changes on coagulation and fibrinolysis markers relative to the lower-dosed COC LNG/EE 100 μg/20 μg^30^. The apparent improved safety profile of E2-containing COCs needs to be confirmed in clinical studies with VTE as endpoint.

COCs containing EE have been associated with pronounced APC resistance^31^-^32^ as well as elevations in procoagulatory factors and reductions in anticoagulatory factors^33^,^34^. Reduced sensitivity for ETP-based APC has been claimed to be associated with VTE risk in men and women, even in the absence of the Factor V Leiden mutation^35^-^36^. In the current study, NOMAC/ E2 resulted in changes in ETP-based APC sensitivity ratios that were substantially and significantly smaller than ratios observed with LNG/EE.

Evidence suggests that the individual composition of different COCs, in terms of hormone dose and progestogen type, also influences their respective effects on lipids and lipoproteins^37^-^38^. Although the changes in lipoprotein metabolism induced by EE are complex, the net effect is generally considered favourable, primarily due to increased HDL concentrations. Progestogens with androgenic properties may reverse the lipid benefits of oestrogen, whereas natural progesterone, progesterone-like derivatives and DRSP may be more likely to preserve these benefits^39^. In the present study, NOMAC/E2 did not induce any meaningful changes in the lipid profile, whereas women receiving LNG/ EE demonstrated a reduction in HDL-C and an elevation in LDL-C and total triglycerides. These results are consistent with those observed in previous studies of LNG/EE^40^-^42^.

Hormonal contraceptives (including COCs) have been associated with subclinical disturbances in carbohydrate metabolism, including impaired glucose tolerance and insulin resistance^43^ that may increase the risk of type 2 diabetes and vascular disease. Studies evaluating the effects of oestrogen on carbohydrate metabolism have reported contradictory findings^44^-^50^. The androgenic properties of the progestogens used in COCs may influence responses to glucose tolerance tests; non-androgenic progestogens appear to exert a neutral effect on carbohydrate metabolism^51^. In the present study, negligible changes in glucose and insulin parameters were observed in women taking NOMAC/E2, whereas significant increases in insulin and glucose AUC_3_ were found in women taking LNG/EE. The increases associated with LNG/EE are in line with those observed in previous studies^40^-^42^, but it is doubtful whether they are clinically meaningful in terms of predicting greater risk.

The role of inflammation in the pathogenesis of cardiovascular disease (CVD) has been increasingly recognised in recent years, and inflammation markers such as the non-specific acute-phase protein CRP have been identified as useful predictors of CVD risk^52^. In clinical studies, elevations in CRP levels have been observed with the use of various COC formulations^53^-^55^. Both NOMAC/E2 and LNG/EE increased CRP levels in this randomised metabolic study, with a significantly greater increase seen with LNG/EE. Whereas the median CRP level observed in the NOMAC/E2 group at cycle 6 (0.70 mg/1) remained well below the threshold indicating increased cardiovascular risk (—2 mg/1)^56^, the median CRP level in the LNG/EE group at cycle 6 (1.70 mg/1) approached this threshold.

SHBG is a carrier protein synthesised in hepato-cytes; its synthesis has been shown to be highly sensitive to oestrogens and androgens^57^. EE administered orally markedly increases SHBG levels in a dose-dependent fashion, whereas progestogens, due to their androgenic properties, decrease these levels to a varying extent depending on the steroid dose and type. The net effect of a COC formulation on SHBG thus appears to reflect the balance between oestrogenic and androgenic activity^57^. In this study, NOMAC/E2 induced an approximate 44% increase from baseline in SHBG, a change that was significantly greater than the 22% rise induced by LNG/EE. However, compared with other COCs, where increases in SHBG vary from 150% for COCs containing norgestimate to 400% for COCs containing cyproterone acetate, the SHBG increases observed in this study were small^57^. The rise in SHBG induced by LNG/EE is most likely the result of the increase due to EE and the decrease due to LNG, a progestogen with residual androgenicity The rise in SHBG induced by NOMAC/E2 is most likely only due to the oestrogen component of this combination because NOMAC is a non-androgenic progestogen.

This is one of the first studies reporting the effect of a COC that contains E2 instead of EE on several metabolic indices. The VTE risk and metabolic effects of the comparator COC LNG/EE (150 Hg/30 μg) have been well established, and it is therefore an appropriate comparator. The influence of NOMAC/E2 on haemostatic indices has also been studied in comparison to the lower-dosed COC containing LNG/EE (100 Hg/20 Hg), with essentially similar results^30^. Although direct comparisons between NOMAC/E2 and COCs containing progestogens other than LNG are not yet available, it is likely that these COCs are associated with greater changes in these indices than NOMAC/E2, in view of their generally more pronounced effects in comparison to LNG/EE^58^,^59^.

The main limitation of this study is the use of surrogate endpoints for metabolic indices. In addition, none of the haemostatic indices measured in this study have been established as definitive markers of thrombosis, and the clinical relevance of the differences found in this study can only be determined by performing large, clinical studies with VTE as endpoint. The same holds true for other indices, including lipids and carbohydrate metabolism.

In summary, NOMAC/E2 had minimal influence on haemostatic, lipid and carbohydrate metabolism indices and caused less change in these parameters than LNG/EE. Large clinical studies will be needed to determine whether these differences in surrogate end-points are also clinically relevant.

## References

[1] http://www.un.org/esa/population/publications/contraceptive2007/7contraceptive_2007_table.pdf.

[2] Rosenberg MJ, Meyers A, Roy V (1999). Efficacy cycle control, and side effects of low- and lower-dose oral contraceptives: A randomized trial of 20 micrograms and 35 micrograms estrogen preparations. Contraception.

[3] Farley TM, Collins J, Schlesselman JJ (1998). Hormonal contraception and risk of cardiovascular disease. An international perspective. Contraception.

[5] Szarewski A, Mansour D, Shulman LP (2010). 50 years of'The Pill': Celebrating a golden anniversary. J Fam Plann Reprod Health Care.

[6] Lidegaard Ø, Lokkegaard E, Svendsen AL, Agger C (2009). Hormonal contraception and risk of venous thromboembolism: National follow-up study. BMJ.

[7] van Hylckama Vlieg A, Helmerhorst FM, Vandenbroucke JP (2009). The venous thrombotic risk of oral contraceptives, effects of oestrogen dose and progestogen type: Results of the MEGA case-control study. BMJ.

[8] Reid RL, Westhoff C, Mansour D (2010). Oral contraceptives and venous thromboembolism consensus opinion from an international workshop held in Berlin, Germany in December 2009. J Fam Plann Reprod Health Care.

[9] Shapiro S, Dinger J (2010). Risk of venous thromboembolism among users of oral contraceptives: A review of two recently published studies. J Fam Plann Reprod Health Care.

[10] Suissa S, Spitzer WO, Rainville B (2000). Recurrent use of newer oral contraceptives and the risk of venous thromboembolism. Hum Reprod.

[11] Dinger JC, Heinemann LA, Kuhl-Habich D (2007). The safety of a drospirenone-containing oral contraceptive: Final results from the European active surveillance study on oral contraceptives based on 142,475 women-years of observation. Contraception.

[12] Hoffmann H, Moore C, Zimmermann H (1998). Approaches to the replacement of ethinylestradiol by natural 17beta-estradiol in combined oral contraceptives. Exp Toxicol Pathol.

[13] Lello S (2010). Nomegestrol acetate: Pharmacology, safety profile and therapeutic efficacy. Drugs.

[14] Sitruk-Ware R (2006). New progestagens for contraceptive use. Hum Reprod Update.

[15] Duijkers IJ, Klipping C, Grab P, Korver T (2010). Effects of a monophasic combined oral contraceptive containing nomegestrol acetate and 17β-oestradiol on ovarian function in comparison to a monophasic combined oral contraceptive containing drospirenone and ethinylestradiol. Eur J Contracept Reprod Health Care.

[16] Christin-Maitre S, Serfary D, Chabbert-Buffet N (2011). Comparison of a 24-day and a 21-day pill regimen for the novel combined oral contraceptive, nomegestrol acetate and 17(3-estradiol (NOMAC/E2): A double- blind, randomized study. Hum Reprod.

[17] Ågren UM, Anttila M, Mäenpää-Liukko K (2011). Effects of a monophasic combined oral contraceptive containing nomegestrol acetate and 17p∼oestradiol in comparison to one containing levonorgestrel and ethinylestradiol on markers of endocrine function. Eur J Contracept Reprod Health Care.

[19] Friedewald WT, Levy RI, Fredrickson DS (1972). Estimation of the concentration of low-density lipoprotein cholesterol in plasma, without use of the preparative ultracentrifuge. Clin Chem.

[20] Gerstman BB, Piper JM, Tomita DK (1991). Oral contraceptive estrogen dose and the risk of deep venous thromboembolic disease. Am J Epidemiol.

[21] Lidegaard Ø, Edström B, Kreiner S (2002). Oral contraceptives and venous thromboembolism: A five-year national case-control study. Contraception.

[22] Jick H, Jick SS, Gurewich V (1995). Risk of idiopathic cardiovascular death and nonfatal venous thromboembolism in women using oral contraceptives with differing progestagen components. Lancet.

[23] Spitzer WO, Lewis MA, Heinemann LA (1996). Third generation oral contraceptives and risk of venous thromboembolic disorders: An international case-control study. Transnational research group on oral contraceptives and the health of young women. BMJ.

[24] Herings RM, Urquhart J, Leufkens HG (1999). Venous thromboembolism among new users of different oral contraceptives. Lancet.

[25] Baillargeon JP, McClish DK, Essah PA, Nestler JE (2005). Association between the current use of low-dose oral contraceptives and cardiovascular arterial disease: A meta-analysis. J Clin Endocrinol Metab.

[26] Lidegaard Ø, Kreiner S (2002). Contraceptives and cerebral thrombosis: A five-year national case-control study. Contraception.

[27] Lindberg UB, Crona N, Stigendal L (1989). A comparison between effects of estradiol valerate and low dose ethinyl estradiol on haemostasis parameters. Thromb Haemost.

[28] Mashchak CA, Lobo RA, Dozono-Takano R (1982). Comparison of pharmacodynamic properties of various estrogen formulations. Am J Obstet Gynecol.

[29] Parke S, Nahum G, Mellinger U, Junge W (2008). Metabolic effects of a new four-phasic oral contraceptive containing estradiol valerate and dienogest. Obstet Gynecol.

[30] Gaussem P, Alhenc-Gelas M, Thomas JL (2011). Haemostatic effects of a new combined oral contraceptive, nomegestrol acetate/17beta-estradiol, compared with those of levonorgestrel/ethinyl estradiol. A double-blind, randomised study. Thromb Haemost.

[31] Rosing J, Tans G, Nicolaes GA (1997). Oral contraceptives and venous thrombosis: Different sensitivities to activated protein C in women using second- and third-generation oral contraceptives. Br J Haematol.

[32] Kemmeren JM, Algra A, Meijers JCM (2004). Effect of second- and third-generation oral contraceptives on the protein C system in the absence or presence of the factor V Leiden mutation: A randomized trial. Blood.

[33] Conard J (1999). Biological coagulation findings in third-generation oral contraceptives. Hum Reprod Update.

[34] Tans G, Curvers J, Middeldorp S (2000). A randomized cross-over study on the effects of levonorgestrel- and desogestrel-containing oral contraceptives on the anticoagulant pathways. Thromb Haemost.

[35] de Visser MC, Rosendaal FR, Bertina RM (1999). A reduced sensitivity for activated protein C in the absence of factor V Leiden increases the risk of venous thrombosis. Blood.

[36] Tans G, van Hylckama Vlieg A, Thomassen MC (2003). Activated protein C resistance determined with a thrombin generation-based test predicts for venous thrombosis in men and women. Br J Haematol.

[37] Godsland IF, Winkler U, Lidegaard Ø, Crook D (2000). Occlusive vascular diseases in oral contraceptive users. Epidemiology, pathology and mechanisms. Drugs.

[38] Godsland IF (2004). Biology: Risk factor modification by OCs and HRT lipids and lipoproteins. Maturitas.

[39] Sitruk-Ware R (2005). Pharmacology of different progestogens: The special case of drospirenone. Climacteric.

[40] Endrikat J, Klipping C, Cronin M (2002). An open label, comparative study of the effects of a dose-reduced oral contraceptive containing 20 microg ethinyl estradiol and 100 microg levonorgestrel on hemostatic, lipids, and carbohydrate metabolism variables. Contraception.

[41] Scharnagl H, Petersen G, Nauck M (2004). Double-blind, randomized study comparing the effects of two monophasic oral contraceptives containing ethinylestradiol (20 microg or 30 microg) and levonorgestrel (100 microg or 150 microg) on lipoprotein metabolism. Contraception.

[42] Skouby SO, Endrikat J, Diisterberg B (2005). A 1-year randomized study to evaluate the effects of a dose reduction in oral contraceptives on lipids and carbohydrate metabolism: 20 microg ethinyl estradiol combined with 100 microg levonorgestrel. Contraception.

[43] Lopez LM, Grimes DA, Schulz KF (2009). Steroidal contraceptives: Effect on carbohydrate metabolism in women without diabetes mellitus. Cochrane Database Syst Rev.

[44] Brussaard HE, Gevers Leuven JA, Frölich M (1997). Short-term oestrogen replacement therapy improves insulin resistance, lipids and fibrinolysis in postmenopausal women with NIDDM. Diabetologia.

[45] Brown MD, Korytkowski MT, Zmuda JM (2000). Insulin sensitivity in postmenopausal women: Independent and combined associations with hormone replacement, cardiovascular fitness, and body composition. Diabetes Care.

[46] Ryan AS, Nicklas BJ, Berman DM (2002). Hormone replacement therapy, insulin sensitivity, and abdominal obesity in postmenopausal women. Diabetes Care.

[47] Howard BV, Hsia J, Ouyang P (2004). Postmenopausal hormone therapy is associated with atherosclerosis progression in women with abnormal glucose tolerance. Circulation.

[48] Os I, Os A, Abdelnoor M (2005). Insulin sensitivity in women with coronary heart disease during hormone replacement therapy. J Womens Health (Larchmt).

[49] Bonds DE, Lasser N, Qi L (2006). The effect of conjugated equine oestrogen on diabetes incidence: The women's health initiative randomised trial. Diabetologia.

[50] Shadoan MK, Kavanagh K, Zhang L (2007). Addition of medroxyprogesterone acetate to conjugated equine estrogens results in insulin resistance in adipose tissue. Metabolism.

[51] Sitruk-Ware R (2000). Progestins and cardiovascular risk markers. Steroids.

[52] Cushman M, Arnold AM, Psaty BM (2005). C-reactive protein and the 10-year incidence of coronary heart disease in older men and women: The cardiovascular health study. Circulation.

[53] van Rooijen M, Hansson LO, Frostegard J (2006). Treatment with combined oral contraceptives induces a rise in serum C-reactive protein in the absence of a general inflammatory response. J Thromb Haemost.

[54] White T, Özel B, Jain JK, Stanczyk FZ (2006). Effects of transdermal and oral contraceptives on estrogen-sensitive hepatic proteins. Contraception.

[55] Johnson JV, Lowell J, Badger GJ (2008). Effects of oral and transdermal hormonal contraception on vascular risk markers: A randomized controlled trial. Obstet Gynecol.

[56] Ridker PM, MacFadyen JG, Fonseca FA (2009). Number needed to treat with rosuvastatin to prevent first cardiovascular events and death among men and women with low low-density lipoprotein cholesterol and elevated high-sensitivity C-reactive protein: Justification for the use of statins in prevention: An intervention trial evaluating rosuvastatin (JUPITER). Circ Cardiovasc Qual Out comes.

[57] Odlind V, Milsom I, Persson I, Victor A (2002). Can changes in sex hormone binding globulin predict the risk of venous thromboembolism with combined oral contraceptive pills?. Ada Obstet Gynecol Scand.

[58] Oral Contraceptive Hemostasis Study Group (2003). The effects of seven monophasic oral contraceptive regimens on hemostatic variables: Conclusions from a large randomized multicenter study. Contraception.

[59] Kluft C, Endrikat J, Mulder SM (2006). A prospective study on the effects on hemostasis of two oral contraceptives containing drospirenone in combination with either 30 or 20 microg ethinyl estradiol and a reference containing desogestrel and 30 microg ethinyl estradiol. Contraception.

